# Can AI Help to Avert the Environmental Great Filter?

**DOI:** 10.1007/978-3-030-65355-2_25

**Published:** 2021-03-20

**Authors:** Eric Postma, Marie Postma

**Affiliations:** 1 Tilburg University, Tilburg, The Netherlands; 2 Tilburg University, Tilburg, The Netherlands; 3 Tilburg University, Tilburg, The Netherlands; 4 Tilburg University, Tilburg, The Netherlands; Department of Cognitive Science and Artificial Intelligence, Tilburg School of Humanities and Digital Sciences, Tilburg, The Netherlands

## Abstract

While the impact of the COVID-19 pandemic on our lives is still evident on a daily basis, there is a much larger disaster looming in our future. We are faced with massive evidence that civilization is threatened by a climate disaster, and drastic measures are needed to avoid a point of no return. Will humankind succeed in adopting the necessary measures in time?

In this essay, we explore the potential of present-day AI systems to mitigate the apparent human inability to respond timely and adequately to the imminent peril threatening the existence of our civilization. We will argue that contrary to focusing on the widespread concerns of AI superseding humanity, the role of AI in climate change solutions needs to be prioritized and appreciated. To illustrate the potential of AI, we first contemplate the suboptimal human response to the nonlinear dynamics of the COVID-19 crisis. Subsequently, we generalize our observations to the climate crisis.

While the impact of the COVID-19 pandemic on our lives is still evident on a daily basis, there is a much larger disaster looming in our future. We are faced with massive evidence that civilization is threatened by a climate disaster, and drastic measures are needed to avoid a point of no return. Will humankind succeed in adopting the necessary measures in time? Some scientists view the possibility of the environmental “Great Filter”—an event that eventually wipes out any instance of intelligent life, including our own—as inevitable (Webb 2002). In the 1950s, the physicist Enrico Fermi, during a lunch with his colleague, famously raised the question “Where are they?” addressing the apparent lack of extraterrestrial intelligent life in a galaxy where the formation of planets is common. For sure, it is a disconcerting idea that the answer to Fermi’s paradox, namely that a Great Filter prevents an expanding lasting life (Hanson 1998), may apply to our own technological society in a not so distant future.

In this essay, we explore the potential of present-day AI systems to mitigate the apparent human inability to respond timely and adequately to the imminent peril threatening the existence of our civilization. We will argue that contrary to focusing on the widespread concerns of AI superseding humanity, the role of AI in climate change solutions needs to be prioritized and appreciated. To illustrate the potential of AI, we first contemplate the suboptimal human response to the nonlinear dynamics of the COVID-19 crisis. Subsequently, we generalize our observations to the climate crisis.

## The COVID-19 Crisis

Similar to the climate crisis, the potential dangers of the coronavirus were known in Europe and the US ahead of the pandemic. Yet, despite the forewarning evidence collected in Wuhan, China, the governmental response in most Western countries was insufficient and overdue. In the famous documentary An Inconvenient Truth, Al Gore used the apt metaphor of a frog in a pot of slowly heated water. Gradually raising the temperature of the water until boiling point leaves the frog inactive until it dies from the heat. At any point, the frog could have jumped out of the pot but it ignores the accumulating evidence.^1^ What the frog fails to acknowledge is the nonlinear dynamics of the boiling event in relation to its ability to save itself, thereby shutting its eyes to the fact that, at some critical point, small changes in temperature parameters will have irrecoverable effects.

**Fig. 25.1:**
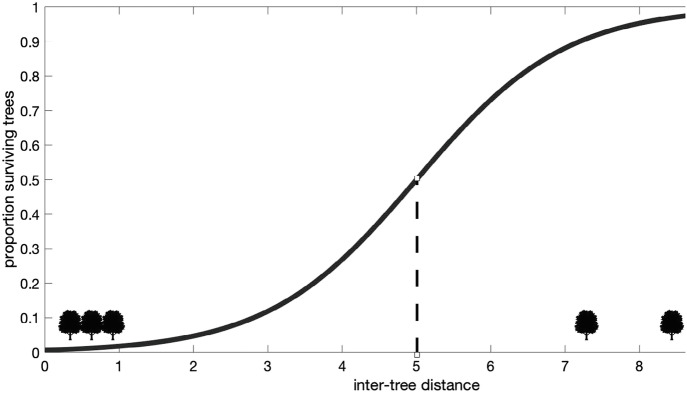
Scenario of a forest fire (source: authors)

The nonlinear dynamics of various events is beautifully captured in the classic model in physics, called the percolation model. The model can be exemplified in the scenario of a forest fire; see Fig. 25.1. Consider a forest in which the trees are positioned at a fixed distance from each other. When the inter-tree distance is large and one tree burns, the other trees remain unaffected. Now imagine a knob (called a parameter in most models) that we can turn to adjust the inter-tree distance. If we turn the knob to a very low inter-tree distance (say, less than 1.5 m), a single burning tree will cause a fire that will devastate the entire forest. The graph shown below sketches the universal pattern witnessed in percolation models. The vertical axis represents the proportion of surviving trees, the horizontal axis the knob position where the inter-tree distance increases by moving to the right along the axis (numbers in arbitrary units). At the value of 5, a so-called “phase transition” occurs. The macroscopic behavior transforms from a disaster into a non-hazardous local fire. Note that the slope of the S-shaped function depends, amongst others, on the size of the forest: for very large forests, the slope becomes much steeper and the phase transition would be very sudden. A very small change in inter-tree distance can then determine the fate of the forest.

Physicists rely on simple models, such as the percolation model because these often exhibit universality. In fact, it is easy to see how the model can be applied to a virus spreading in the human population, with a small change in social distancing measures resulting in either halting or accelerating the pandemic. Unfortunately, our high-school mathematics curriculum is mainly focused on linear relationships: small changes in a parameter value induce small changes in the output value. Consequently, we often lack intuition for nonlinear behaviors (May 1976) and implicitly assume that small changes have small effects.

In the case of pandemics, the expectation of a linear growth may lead to a dangerous underestimation of the speed with which the disease can spread in a population. According to Zacks and Franconeri (2020), even when confronted with nonlinear simulations, people will find the nonlinearity counterintuitive and have the tendency to extrapolate a straight growth line. The importance of integrating reliable data with appropriate models (Alamo et al. 2020) is demonstrated by the analysis of the response of the Dutch Government to the COVID-19 pandemic. The Dutch national newspaper NRC Handelsblad (NRC 2020) described how a microbiologist from the city of Breda, Jan Kluytmans, initiated the introduction of an intelligent lockdown. On March 22, 2020, Kluytmans used a British epidemiological model from the internet to compute the number of infected persons, given the 18 COVID-19 fatalities in the province of North-Brabant. Whereas the RIVM estimate for March 22 was 1413 COVID-19 infections, the computation performed by Kluytmans resulted in a much larger number: at least 42,000 infections two-and-half weeks prior to March 22. With so many infections two-and-half weeks prior to March 22, the number of infections should be certainly higher than the RIVM estimate of 1413. Kluytmans’s intervention may have averted a disaster in the Netherlands; in terms of the percolation model, we may have been near the phase transition point.

## The Climate Crisis and Hybrid Intelligence

The climate crisis is comparable to the COVID-19 crisis, albeit at a more moderate timescale. Our civilization as we know it is unsustainable. Unprecedented increases in global temperature and pollution levels represent warning signals that we are aware of, yet a collective action at the governmental level has been slow, insufficient, and mired by short-term political gains. AI is not a panacea for climate change, but it can positively influence and guide the transition towards a sustainable society.

First and foremost, AI algorithms can be used to improve the predictions of climate models (Huntington et al. 2019). By solving discrepancies in the outputs of currently employed models, such as the Earth System Model (ESM), and by refining their estimates, climate adaptation planning can be improved. In addition, AI algorithms can facilitate the measurement of environmental factors. An example of how this can be done is an AI algorithm we recently developed to quantify automatically floating plastic waste using video cameras on bridges (van Lieshout et al. 2020).

A second major target is the transformation of the information context into a sustainable one while avoiding the anti-democratic pitfalls of persuasive computing, i.e., the use of technology to steer an individual course of action (Helbing et al. 2017; Postma 2019). In her recent paper “Should AI be Designed to Save Us from Ourselves?,” Lahsen (2020) states that “[o]nly rethinking and redesign of the principles and technologies that generate information contexts within which public decisions are made can positively engender the future we will get” (p. 66). Innovations in AI should focus on facilitating the creation of such information contexts in order to overcome the human tendency to forage information that leads to the desirable conclusion rather than an undesirable belief (Kruglanski et al. 2020). Ironically, AI algorithms are currently used on a massive scale by technology companies to create information contexts that may cater to prior beliefs yet lack long-term viability. The challenge is to counter this trend with AI technologies specifically designed to support the transition to a sustainable society. One example of how this can be done is provided by Rolnick et al. (2019) who present an impressive overview of ways in which AI and machine learning can tackle climate change. An important tool for individual action is the use of AI to provide individuals with instantaneous feedback about their carbon footprint. Such personalized feedback shapes our decisions about modes of transport, types of diets, and purchases. Realizing AI-supported personalized feedback systems coupled to financial incentives (motivation) has the potential to facilitate behavior change.

## How to Avoid the Entropic Abyss: A Case for Hybrid Intelligence

Despite the clear wins that the use of sophisticated AI models can deliver in our struggle to avoid the planetary entropic abyss, it is important to remember that, while AI systems based on machine learning are very good at making predictions, they lack common sense reasoning. For instance, if there were a sudden surge in hospitalizations due to an extremely cold weather period, any human expert would understand that this may confound the COVID-19 prediction model. On the other hand, AI models may overcome the limited information-processing capacity of humans by taking into account all relevant data. Clearly, at the current stage of AI research, the best of both worlds is the combination of human and machine intelligence, so-called “hybrid intelligence.”

Hybrid intelligence aims at expanding human intelligence instead of replacing it. It offers the best of both worlds by combining the general intelligence of humans with the narrow task-specific intelligence of AI. The development of hybrid intelligence requires the knowledge of experts who understand both humans and machines, including their strengths and weaknesses. The unique Tilburg University research and education program Cognitive Science and AI represent a hotspot of such interdisciplinary talent. In the end, whether the age of Anthropocene will lead to collapse or to the metaphorical frog being rescued will depend on our society making—or being nudged to make—that decision. It is highly unlikely that the new common can be achieved without the help of AI technology that understands and supplements human cognition and motivation.

## Footnotes

1 For your information, the metaphor is based on an incorrect assumption (Fallows 2006). According to biologists, a frog that is gradually heated in a pot will actually jump out on time, thereby apparently demonstrating a better survival instinct than the human species.
